# Integrated multi-omics analysis reveals key hub genes and mechanisms in calcific aortic stenosis

**DOI:** 10.3389/fcvm.2025.1640014

**Published:** 2025-11-14

**Authors:** Feng-Xia Wang, Shuai Liu, Hao-Qiang Guo, Jia-Qing Yu, Jun Cui, Qi Cheng, Nilupaer Aisikeer, Fen Liu, Ming-Jun Duan, Xiang Xie, Yi-tong Ma

**Affiliations:** 1 First Affiliated Hospital of Xinjiang Medical University, Urumqi, Xinjiang, China; 2 Department of Human Anatomy, School of Basic Medical Sciences, Xinjiang Medical University, Urumqi, Xinjiang, China; 3 Animal Experimental Center, Xinjiang Medical University, Urumqi, Xinjiang, China; 4 Xinjiang Key Laboratory of Cardiovascular Disease Research, Urumqi, Xinjiang, China

**Keywords:** aortic stenosis, calcification, transcriptome, proteome, single-cell transcriptomics

## Abstract

**Objective:**

Aortic stenosis (AS) is a critical risk factor for the development of structural heart disease, and identifying its pathogenic genes will provide new insights into cardiac pathology and treatment.

**Methods:**

“edgeR” was used to calculate differentially expressed genes (DEGs) for bulk-RNAseq. GO, KEGG, and GSEA analyses were performed on the DEGs. Aortic valves from 8 AS patients and 8 non-AS patients were collected for proteomic sequencing. After DEG analysis, five algorithms were used to identify hub genes. ROC curves were constructed for the hub genes. Single-cell RNA sequencing (scRNAseq) was applied to systematically elaborate the mechanism in AS pathogenesis.

**Results:**

Transcriptome data showed that AS was accompanied by high expression of genes such as MMP9, CXCL8, and SPP1, with significant activation of hypoxia, inflammatory response, and fibrosis. Proteomic sequencing of calcified AS revealed significantly enhanced hypoxic response, TNF-α signaling, and extracellular matrix (ECM) formation. Sixteen hub genes, including ITGB3, ITGAV, and MMP9, were identified by five algorithms, all with high diagnostic efficacy (AUC > 0.75). PCR experiments confirmed that MMP9 and PLAU were highly expressed in calcified aortic valves (*P* < 0.05). scRNAseq revealed that in highly calcified regions, MMP9 and PLAU were mainly distributed in endothelial cells, monocytes, and macrophages, participating in the differentiation of monocytes and macrophages and relating to lipid metabolism and proinflammatory responses.

**Conclusion:**

The 16 hub genes can assist in the diagnosis of aortic stenosis, and MMP9 and PLAU may participate in AS development by regulating the proinflammatory effects of monocytes and macrophages.

## Introduction

Aortic stenosis (AS) is a cardiac valvular disease caused by structural abnormalities of the aortic valve, leading to left ventricular outflow tract obstruction, primarily manifested as leaflet thickening, calcification, and limited mobility. In structural heart diseases, AS accounts for approximately 25%–30%, with a significantly increasing prevalence with age—the prevalence of severe AS in individuals over 75 years old reaches 3%–5% (1). The 5-year mortality rate of untreated severe AS patients exceeds 50%, and the 2-year mortality rate is as high as 50%–80% when combined with heart failure symptoms (2). Its pathological features include valvular fibrocalcification (calcium deposition in leaflets and annulus), congenital bicuspid aortic valve malformation (accounting for 30%–50% of AS cases), and inflammation-mediated extracellular matrix remodeling (3). Major risk factors include age, bicuspid aortic valve, hypertension, hyperlipidemia, chronic kidney disease, and metabolic syndrome (4).

The etiology of AS is complex, involving multi-level pathological mechanisms. At the tissue level, degenerative calcification is the most common cause (accounting for >80% of elderly patients), characterized by rupture of leaflet collagen fibers, lipid deposition, and abnormal aggregation of hydroxyapatite crystals, leading to valve thickening and stiffness (5). Congenital bicuspid aortic valve is an important inducer (30%–50% of AS cases), whose abnormal blood flow shear stress accelerates valve fibrosis and calcification (4). Stimulated by inflammatory factors and oxidative stress, valvular interstitial cells differentiate into osteoblast-like cells by activating the Runx2/BMP2 signaling pathway, promoting calcium nodule formation (6). Additionally, macrophage infiltration releases matrix metallo-proteinases (MMPs) that degrade the extracellular matrix (ECM), further exposing calcification sites (7).

Calcification in AS primarily occurs on the ventricular side of the leaflets and the fibrosa layer, with its distribution closely related to local biomechanics and molecular microenvironment. The latest histopathological study (8) shows that AS calcification originates in the collagen fiber rupture zone of the fibrosa layer, then spreads along stress-concentrated regions (ventricular side), forming multifocal hydroxyapatite deposits. High-resolution micro-CT reveals that calcification density on the ventricular side is 3–5 times higher than that on the aortic side. Especially in patients with bicuspid aortic valve (BAV), abnormal blood flow shear stress directly enhances endothelial injury on the ventricular side and activates the osteogenic phenotype of valvular interstitial cells (VICs) (9). Single-cell RNA sequencing further reveals that ventricular-side VICs highly express osteogenic differentiation markers (such as RUNX2, BMP2), accompanied by macrophage infiltration releasing IL-1β and TGF-β to drive the fibrocalcification cascade (10). Additionally, studies based on hydrodynamic simulations indicate that the ventricular side, subject to higher cyclic tensile stress (>50 kPa), promotes the expression of calcification-related genes through the integrin-ERK1/2 pathway (11).

This study first performed a combined analysis of RNA-seq data from two groups of aortic calcification patients, collected clinical patient samples for proteomic sequencing, identified hub genes using five algorithms and combined them with RNA-seq analysis, and finally used single-cell transcriptome sequencing data to explore the mechanism by which genes participate in the occurrence of aortic calcification.

## Methods

### Transcriptome data download and preprocessing

GSE51472 and GSE12644 were downloaded from the GEO database (12, 13). GSE51472 included 5 control, 5 sclerotic, and 5 calcified samples, while GSE12644 included 10 control and 10 calcified samples. In R software, Counts data were converted to FPKM and then log-normalized. Sample boxplots were plotted to assess the degree of normalization.

### Differentially expressed gene analysis and GSEA

“edgeR” (14) was used to calculate gene expression changes, and DEGs were screened with the threshold of Log2|FC| ≥ 1 and adjusted *P*-value <0.05. “msigdbr” (15) was used for gene set enrichment analysis (GSEA) of DEGs, and “enrichplot” was used to plot the top-ranked terms.

### PPI network construction, GO and KEGG analysis

The STRING database (16) was used to construct the protein-protein interaction (PPI) network of DEGs, and Cytoscape software (17) was used to visualize the interaction relationships between genes. “clusterProfiler” (18) was used for GO and KEGG pathway analysis using a significance cutoff of *P* < 0.05, and the SRPLOT platform (19) was used to visualize the relevant enriched terms.

### Patient sample collection and proteomic sequencing

The aortic valve tissues of patients with aortic regurgitation (control) and AS in the hospital from January to April, 2024 were collected. Among them, the organization acquisition method is implemented in accordance with relevant guidelines and regulations, and it is confirmed that all subjects and/or their legal guardians have obtained informed consent. This project was approved by the Xinjiang Uygur Autonomous Region People's Hospital (KY2024030102). Aortic valve tissues were washed with pre-cooled saline within 10 min to remove blood residues. Leaflets were separated, tissues were cut into small pieces (<0.2 cm^3^), snap-frozen in liquid nitrogen, and stored at −80 °C. After thawing, tissues were soaked in decalcification solution (4 °C, 24–48 h), with fresh solution replaced every 6 h. After decalcification, tissues were ground into powder with liquid nitrogen, and interference was removed by differential centrifugation. Protein expression was detected by liquid chromatography-mass spectrometry (LC-MS/MS), and MaxQuant was used to match mass spectrometry data to the protein database.

### HE staining and alizarin Red staining

The HE staining procedure for aortic valve tissues included: formalin fixation for 24–48 h, dehydration (gradient ethanol treatment), transparency (xylene), paraffin embedding, and sectioning; the staining process included dewaxing and rehydration, hematoxylin nuclear staining for 5–10 min, hydrochloric acid-ethanol differentiation, water reblueing, eosin cytoplasm staining for 1–2 min, followed by gradient ethanol dehydration, xylene transparency, and neutral gum sealing for microscopic observation of cell morphology and collagen fiber structure.

The alizarin red staining procedure for aortic valve tissues was: dewaxing sections to water, staining in alizarin red S solution for 5–10 min, washing with running water to remove floating color; counterstaining nuclei with hematoxylin for 30 s, hydrochloric acid-ethanol differentiation, water reblueing, gradient ethanol dehydration, xylene transparency, and neutral gum sealing.

### Identification of key gene modules and Hub genes

The MCODE algorithm (20) was used to identify key modules in the PPI network. Five algorithms in Cytohubba (21) were used to detect the top 30 key genes in the PPI network. UpSet (22) was used to visualize the overlap of the five algorithms.

### ROC curve, transcriptional regulation, and m6A modification prediction

The SRPLOT platform was used to construct ROC curves for proteomic sequencing data. The TRRUST database (23) was used to predict transcription factors of hub genes, and the M6A2Target database (24) was used to predict m6A-modified genes of hub genes.

### PCR experiments

PCR experiments were performed to detect the mRNA expression levels of MMP9 and PLAU in aortic valve tissues. Specific steps: frozen tissues were ground in liquid nitrogen, lysed using an RNA extraction kit, centrifuged to remove impurities, and total RNA was purified by binding to an RNA adsorption column. Reverse transcription was performed according to the Takara PrimeScript RT Master Mix instructions (42 °C for 15 min, 85 °C for 5 s to inactivate), synthesizing cDNA; qPCR amplification was performed using Takara SYBR Premix Ex Taq (95 °C pre-denaturation for 30 s, 40 cycles: 95 °C for 5 s, 60 °C for 30 s). Melting curves were used to verify product specificity, and the relative expression of target genes was calculated. The primer sequences as shown in Table 1.

**Table 1:** Information on gene primer sequences.

Gene	Forward primer	Reverse primer
*GAPDH*	5′-ACACCCACTCCTCCACCTTTG-3′	5′-TCCACCACCCTGTTGCTGTAG-3′
*MMP9*	5′-GGCACCACCACAACATCACC-3′	5′-GGGCAAAGGCGTCGTCAATC-3′
*PLAU*	5′-GGCTTAACTCCAACACGCAAGG-3′	5′-AACGGATCTTCAGCAAGGCAATG-3′

### Single-cell transcriptome data preprocessing and DEG analysis

Published single-cell transcriptome data (GSE220774) (25) from aortic calcification patients were collected, including single-cell transcriptome data from three regions (fibrosa layer, ventricular layer, and intermediate layer/remaining layer) of five patients. Data preprocessing strictly followed the Seurat official recommended pipeline (26), including filtering low-quality cells and noise genes, data normalization, identification of highly variable genes, principal component analysis for dimensionality reduction, Louvain clustering algorithm for cell subset identification, cell type annotation using “SingleR” and “Cellmarker” (27, 28), and finally “FindMarkers” for DEG analysis between different cell populations.

### Cell pseudotime analysis

Cell pseudotime analysis maps single-cell transcriptome data to a low-dimensional space, constructs developmental or differentiation trajectories between cells, and infers dynamic changes in cell states. Monocle3 (29) was used to analyze the differentiation trajectories of monocytes and macrophages, which assigns a “pseudotime” value to each cell, identifies differential gene modules along the trajectory, reveals differentiation-driving genes and branching events, and finally visualizes time-dependent gene expression patterns through trajectory plots. The specific steps include using DDRTree to reduce dimensionality, sort and map cells, and the built-in Branched expression analysis modeling (BEAM) is used to assist in branch judgment.

## Results

### Activation of inflammatory and fibrosis in aortic sclerosis and calcification

**Figure 1:**
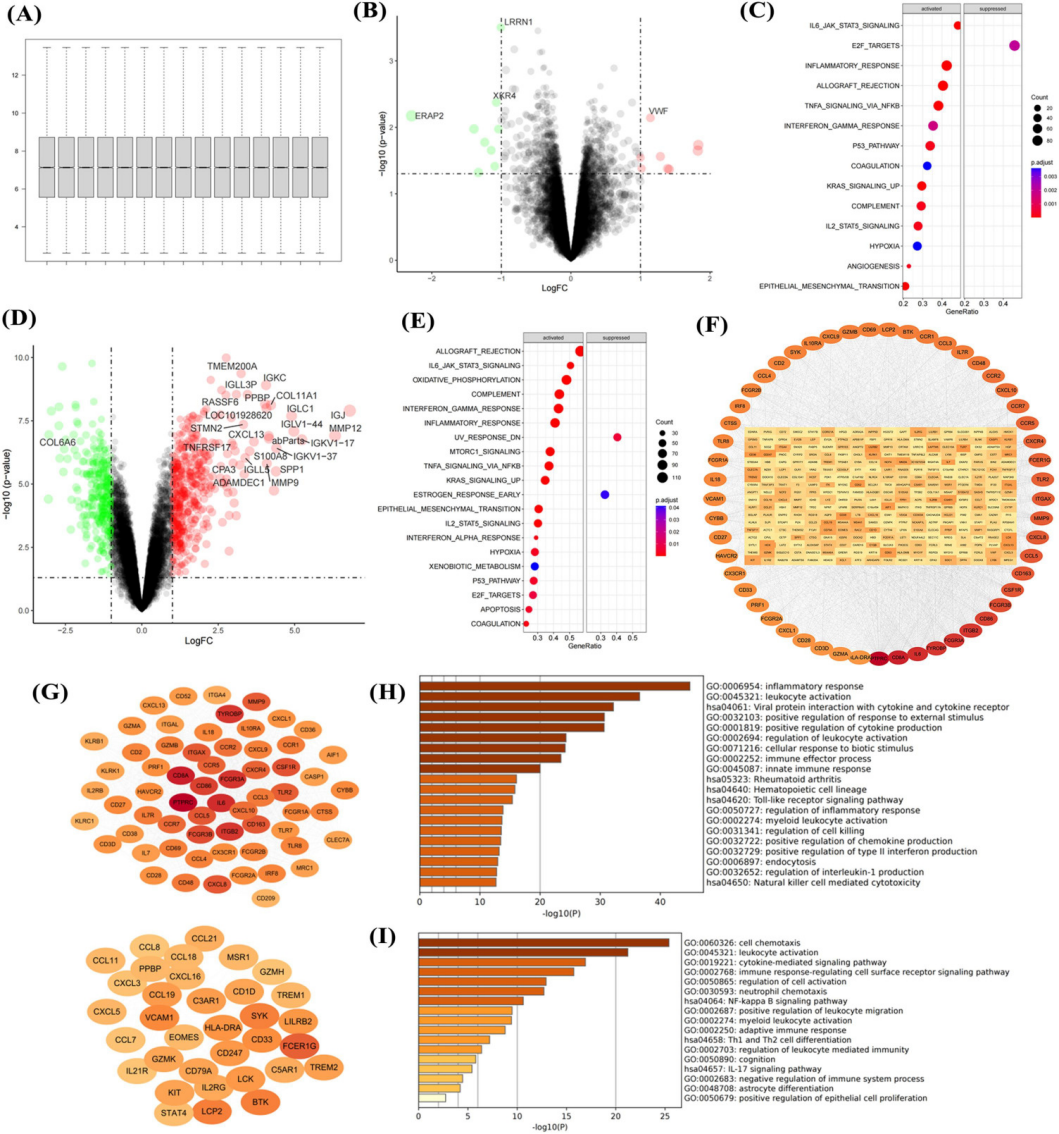
Transcriptional dysregulation in aortic calcification. Sample normalization of the GSE51472 dataset **(A)**, DEGs in aortic sclerosis patients compared with controls **(B)**, and gene set enrichment analysis **(C)**; DEGs in aortic calcification patients **(D)**, GSEA **(E)**, PPI network **(F)**, networks of the top two key gene modules **(G)**, and enrichment analysis of key modules (H,I). Sample size: Control group (*n* = 5); AS group (*n* = 5).

After gene annotation and normalization of the GSE51472 dataset (Figure 1A), comparison of gene expression between the control group and aortic sclerosis group showed that aortic sclerosis had minimal impact on gene expression (Figure 1B), but activated proinflammatory signals (IL-6-STAT3, TNF-α, and IL-2-STAT5), hypoxic signals, and fibrosis (epithelial-mesenchymal transition) (Figure 1C). Compared with the control group, aortic calcification patients had significantly upregulated collagen molecules (COL1A1 and SPP1), proinflammatory molecules (CXCL13, TNFRSF17, and S100A8), and matrix metalloproteinase (MMP) family genes (Figure 1D). Meanwhile, GSEA results for aortic calcification and sclerosis were consistent (Figures 1C,E), and the PPI network of upregulated genes showed that integrins and proinflammatory factors played important roles (Figure 1F). Two key modules were identified by the MCODE algorithm (Figure 1G), both related to inflammatory responses such as cytokine production, Toll-like receptor pathway, cell chemotaxis, and NF-*κ*B signaling pathway (Figures 1H,I). This suggests that the accumulation of extracellular matrix (ECM) and the local inflammatory microenvironment may jointly promote the formation of aortic calcification.

### Upregulation of cell chemotaxis and ECM formation in aortic calcification

**Figure 2:**
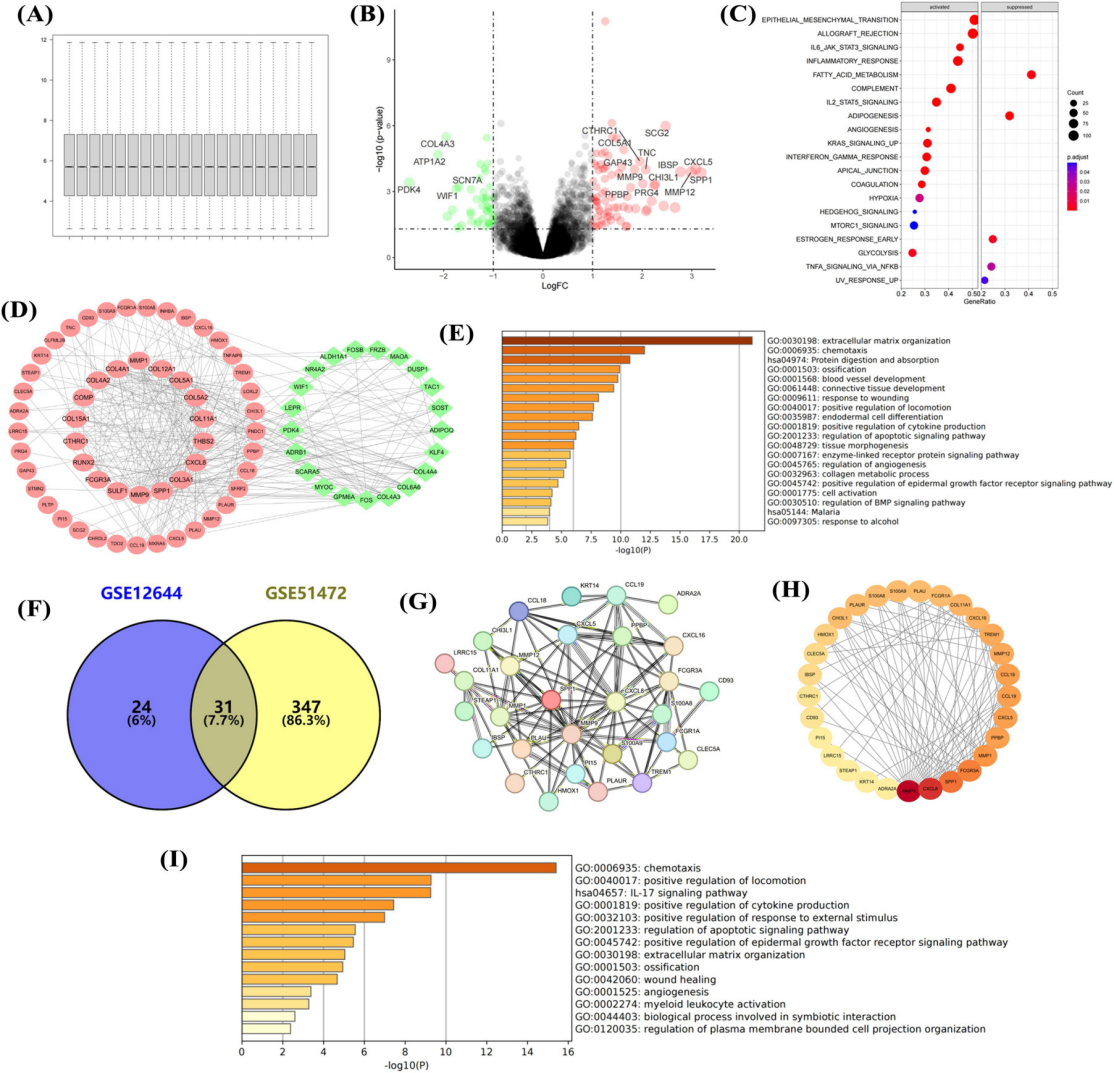
Transcriptional dysregulation in aortic calcification. Sample normalization of the GSE12644 dataset **(A)**, DEGs in aortic calcification patients compared with control group **(B)**, GSEA **(C)**, PPI network **(D)**, and GO/KEGG enrichment analysis **(E)**; identification of intersection genes, PPI network (G,H), and GO/KEGG enrichment analysis **(I)**. D: Red for upregulated genes, green for downregulated genes; H: Ranked by Degree, with darker colors indicating higher ranks. Sample size: Control group (*n* = 10); AS group (*n* = 10).

Normalization of the GSE12644 dataset (Figure 2A) showed that aortic calcification significantly increased genes such as MMP9, MMP12, and SPP1 compared with the control group (Figure 2B), which are involved in inflammatory response and fibrosis progression (Figure 2C). Construction of the PPI network revealed that upregulated MMP9, SPP1, and COL3A1 were in central positions (Figure 2D). Similar to the GSE51472 dataset, these DEGs were mainly related to extracellular matrix formation, cell chemotaxis, and cytokine production (Figure 2E). Intersection analysis of the two datasets identified 31 genes significantly upregulated in aortic calcification (Figure 2F), with MMP9, CXCL8, SPP1, and PLAU ranking among the top (Figures 2G,H). GO and KEGG enrichment analyses showed that intersection genes were related to cell chemotaxis and ECM formation, consistent with the pathological changes in the overall valve tissue (Figure 2I).

### Collection of as patients and proteomic sequencing

**Figure 3:**
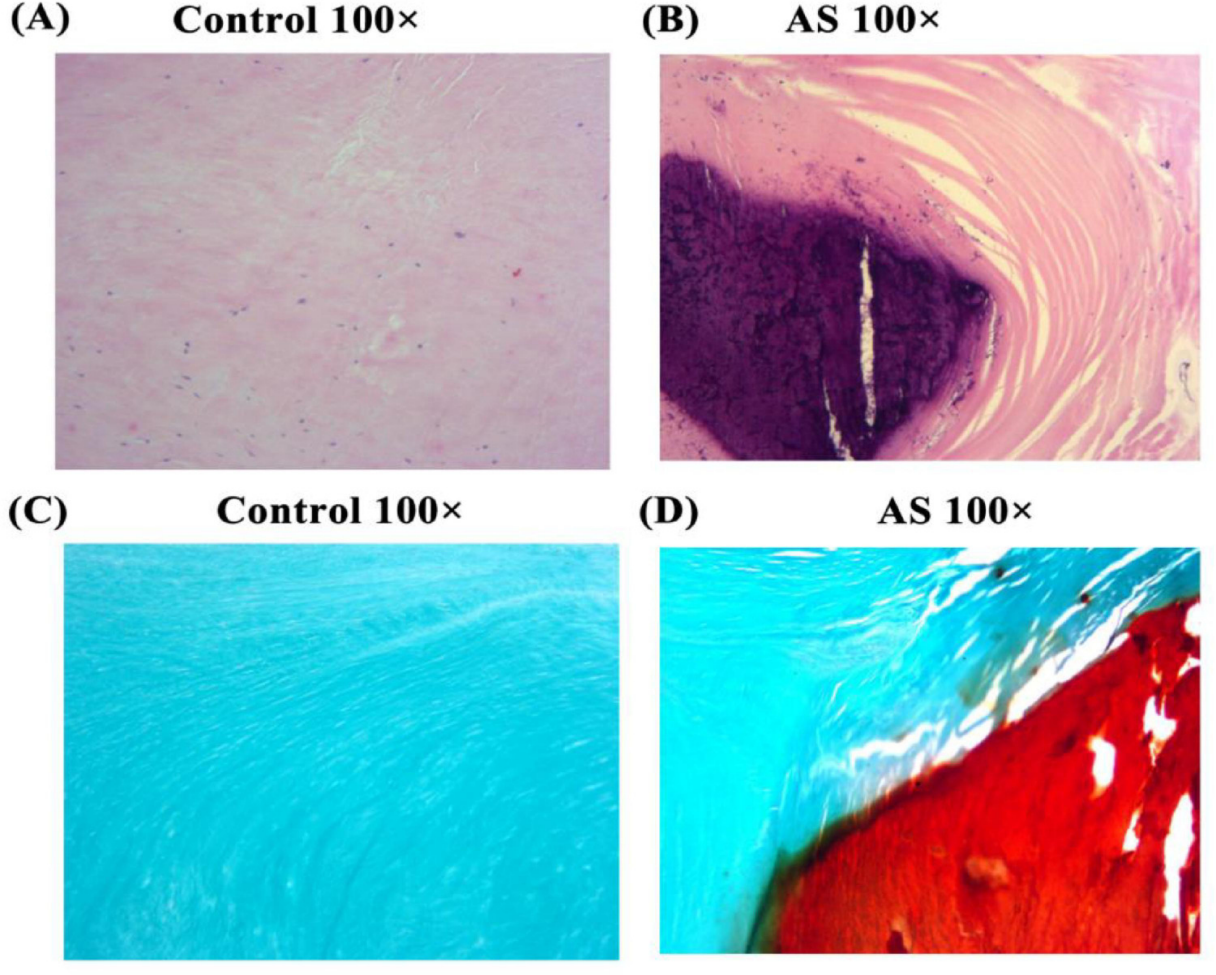
He and alizarin red staining. HE and alizarin red staining of tissues from non-stenotic **(A,C)** and stenotic **(B,D)** aortic valves.

Eight aortic valves from patients with aortic regurgitation (control) and eight from AS patients were collected, with basic information listed in Supplementary Table S1. HE staining of valve tissues showed that collagen fibers (red) in the control group were neatly arranged at 100× magnification (Figure 3A), while those in AS patients showed disorganized collagen fibers with extensive blue-violet calcium salt deposition (Figure 3B). Alizarin red staining showed that normal valve tissues had almost no red staining and aggregation (Figure 3C), while AS valves had abundant red complexes with minimal adhesion at junctions (Figure 3D). Aortic calcification is accompanied by the activation of tissue fibrosis, yet its driving factors remain to be comprehensively evaluated.

### Proteomic characteristics of AS

**Figure 4:**
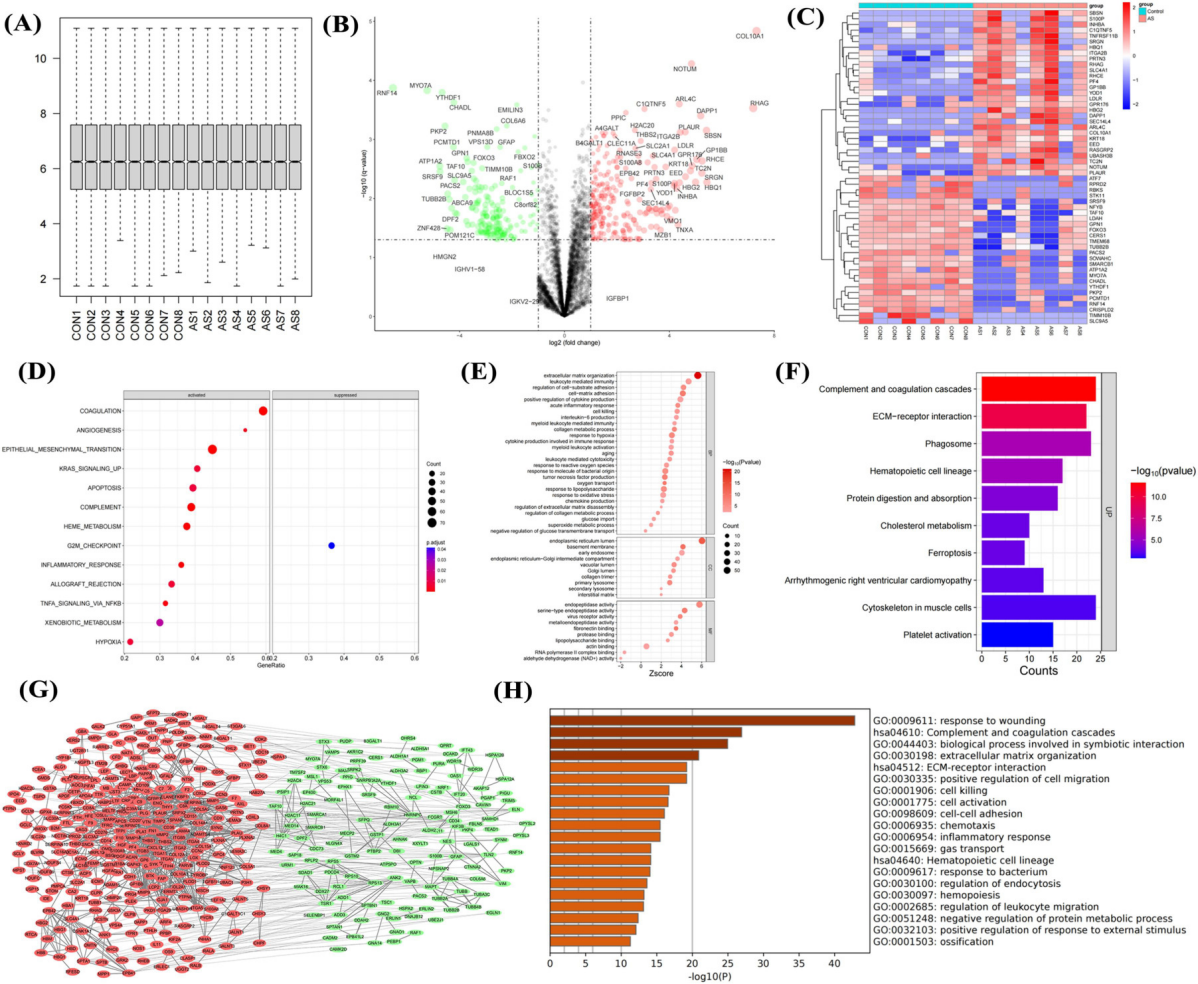
Proteomic sequencing analysis of aortic valves from non-stenotic and stenotic patients. Quality control **(A)**, volcano plot **(B)** and heatmap **(C)** of DEGs, GSEA **(D)**, GO **(E)** and KEGG analysis **(F)**, PPI network **(G)**, and enrichment analysis of upregulated genes **(H)** Sample size: Control group (*n* = 8); AS group (*n* = 8).

Since proteins are the primary executors of cellular functions, proteins were collected and subjected to proteomic sequencing in this study. After proteomic sequencing of 16 samples, gene annotation and normalization were performed (Figure 4A). DEGs showed significant upregulation of proteins such as COL10A1, THBS2, and S100A8 (Figure 4B). Heatmaps showed stable high expression of COL10A1, S100P, and ITGA2B in AS (Figure 4C), with these DEGs involved in inflammatory response, hypoxia, and fibrosis (Figure 4D), consistent with transcriptomic data. GO enrichment analysis showed these genes were related to ECM formation, interleukin and chemokine production (Figure 4E), as well as pathways such as complement and coagulation cascades, and ECM-receptor interaction (Figure 4F). The PPI network showed that dysregulated genes were primarily upregulated (Figure 4G), participating in processes such as wound healing response, ECM formation, and cell chemotaxis (Figure 4H). This further confirms that immune cell activation and fibrosis are risk factors for aortic calcification.

### Identification and expression validation of hub genes

**Figure 5:**
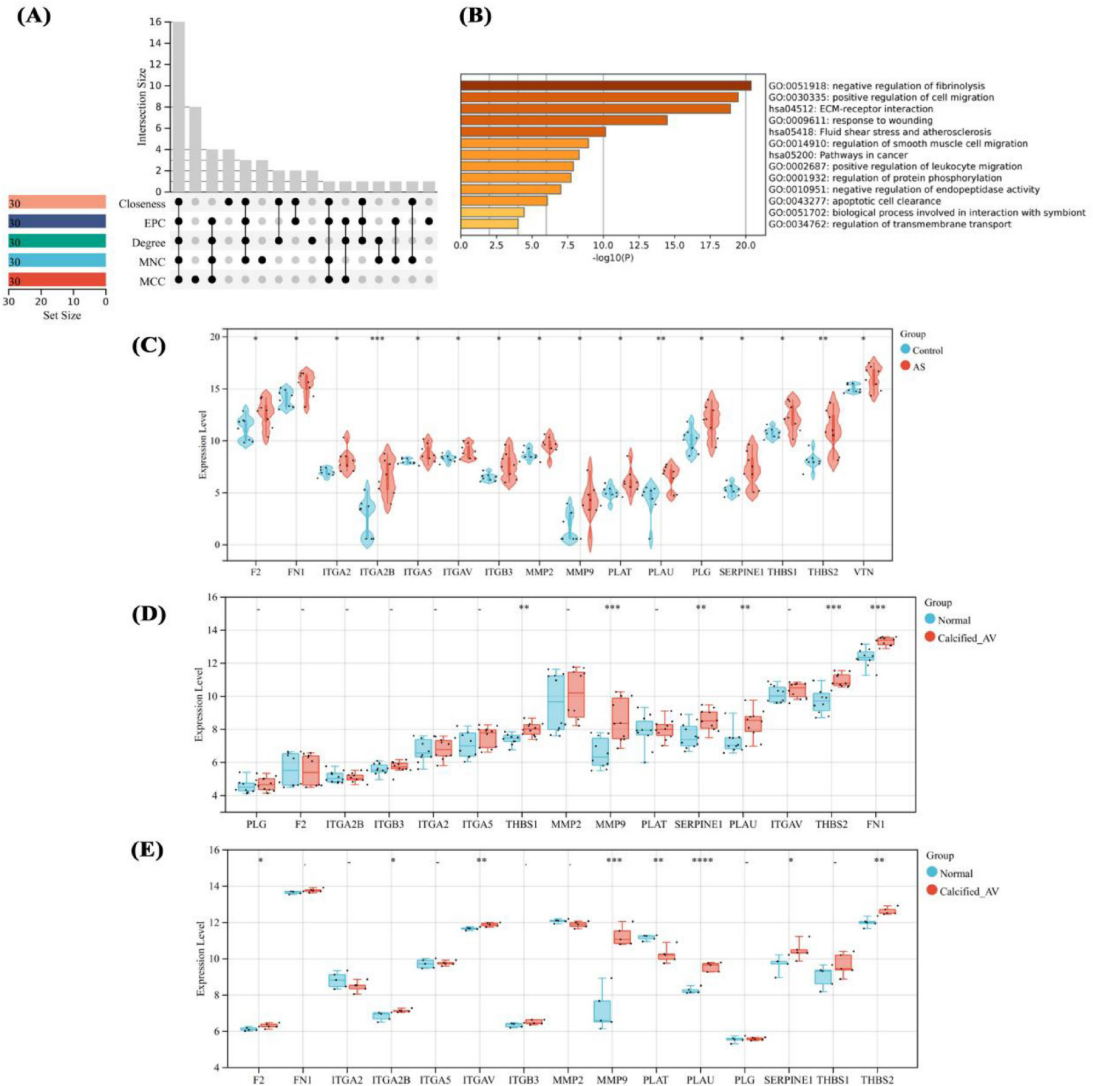
Identification and expression analysis of hub genes. Hub gene identification by multi-algorithms **(A)**, enrichment analysis of hub genes **(B)**, expression analysis of hub genes in proteomic sequencing **(C)**, and their expression in GSE12644 and GSE51472 datasets **(D,E)**. differences in mean values between the control group and AS group were analyzed using an independent samples *t*-test. Statistical significance was set at *P* < 0.05.

To identify the driving factors that drive aortic calcification, five algorithms were used to calculate the top 30 genes in the DEG network, with overlapping genes defined as hub genes. Sixteen hub genes were obtained (Figure 5A), significantly enriched in processes such as ECM-receptor interaction, damage response, and leukocyte migration (Figure 5B). Analysis of hub gene expression in proteomic data showed upregulation in AS (Figure 5C), while in datasets GSE12644 and GSE51472, only MMP9, PLAU, THBS2, and SERPINE1 had significantly increased mRNA expression in calcified aortic valves (*P* < 0.05, Figures 5D,E).

### Diagnostic efficacy of hub genes and prediction of gene regulatory network

**Figure 6:**
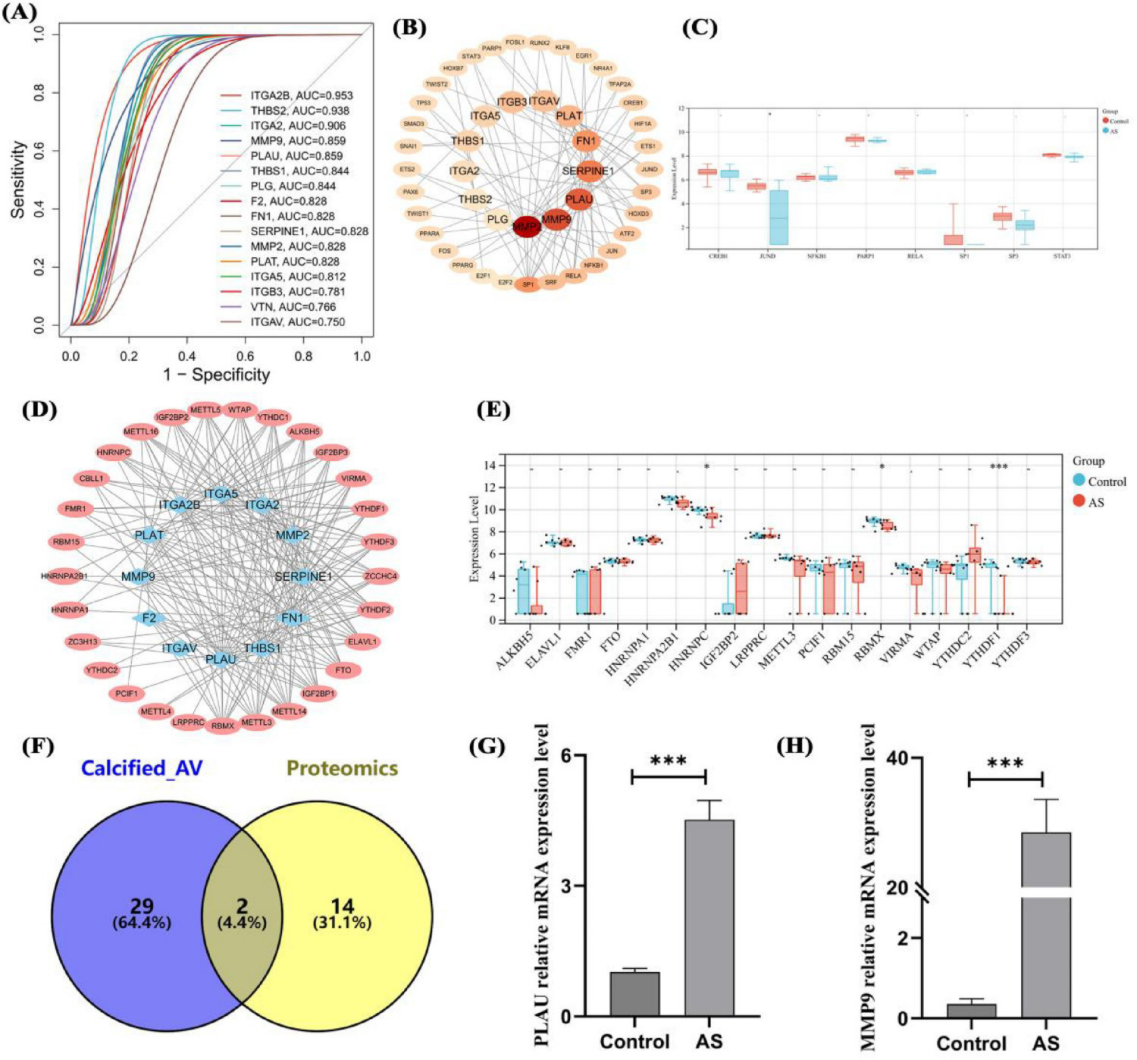
Diagnostic efficacy, transcriptional regulation, RNA modification, and expression validation of hub genes. ROC curves of hub genes in proteomics **(A)**, transcriptional regulatory network **(B)** and expression analysis **(C)** of hub genes, m6A modification **(D)** and expression analysis **(E)** of hub genes, intersection analysis of transcriptomic DEGs and hub genes **(F)**, and mRNA expression validation of overlapping genes (G,H). Sample size: Control group (*n* = 8); AS group (*n* = 8). differences in mean values between the control group and AS group were analyzed using an independent samples *t*-test. Statistical significance was set at *P* < 0.05.

To clarify the important value of the identified genes, 16 hub genes were predicted AS in proteomic data. 13 hub genes including ITGA2B, THBS2, and MMP9 had ROC values >0.8, indicating good diagnostic efficacy in distinguishing AS (Figure 6A). To clarify the regulation of hub genes, transcription factors were predicted, identifying 33 TFs with regulatory relationships to hub genes (Figure 6B), but these TFs had no impact on the high expression of hub genes (Figure 6C). m6A modification prediction showed that hub genes such as MMP9 and PLAU were regulated by 29 m6A enzymes (Figure 6D), with significantly reduced protein expression levels of RBMX, YTHDF1, and HNRNPC (*P* < 0.05, Figure 6E). Intersection of aortic calcification intersection genes (transcriptome) and hub genes (proteome) yielded two genes, PLAU and MMP9 (Figure 6F). qPCR results showed significantly higher mRNA expression of PLAU and MMP9 in AS compared with controls (Figures 6G,H).

### Expression analysis of genes in different cells of aortic calcification patients

**Figure 7:**
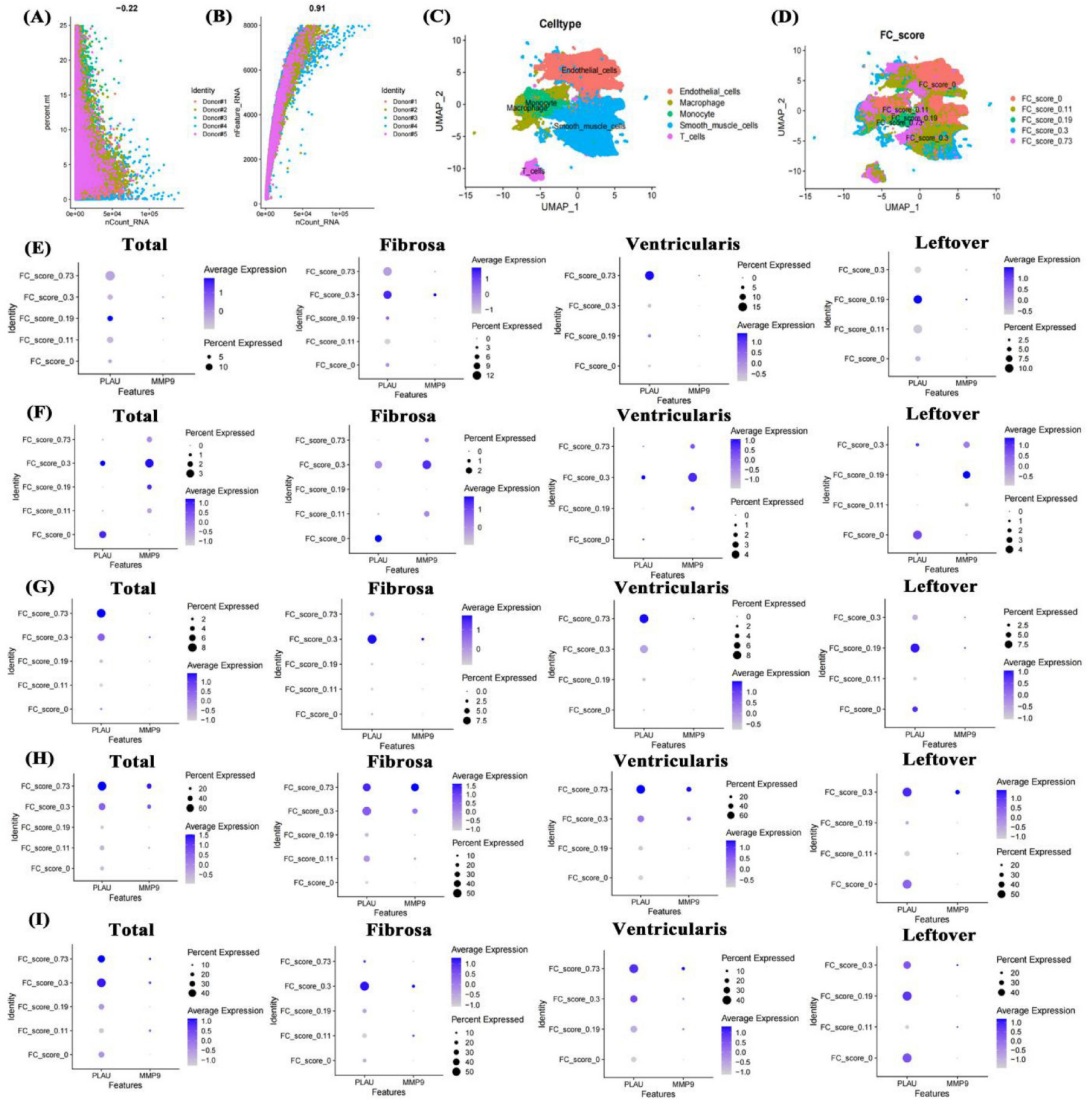
Single-cell transcriptome data analysis of valve calcification patients. Data quality control **(A,B)**, UMAP of cell annotation and calcification scores **(C,D)**, expression of PLAU and MMP9 in smooth muscle cells, T cells, endothelial cells, macrophages, and monocytes across different FC scores and positions **(E–I)**. FC score: fibro-calcification score.

To clarify the molecular mechanism of PLAU and MMP9 in aortic calcification, published patient single-cell transcriptome data (GSE220774) were collected and characterized. In sequencing data, the number of RNAs showed no significant correlation with mitochondrial proportion (Figure 7A) but a high correlation with RNA features (Figure 7B), indicating high data quality. Cell annotation identified endothelial cells, macrophages, monocytes, smooth muscle cells, and T cells (Figure 7C), distributed across different fibro-calcification (FC) scores (Figure 7D). In total smooth muscle cells, PLAU and MMP9 expression had no obvious correlation with FC scores, with PLAU mainly highly expressed in ventricular-side smooth muscle cells of highly calcified regions (Figure 7E). In both overall and region-specific T cells, the two genes were mainly expressed in T cells of moderately calcified regions (Figure 7F). In endothelial cells, PLAU was highly expressed in ventricular-side endothelial cells of calcified regions (Figure 7G). In both overall and regional analyses, PLAU and MMP9 were significantly highly expressed in macrophages of highly calcified regions (Figure 7H), and monocytes, similar to endothelial cells, had PLAU highly expressed in ventricular-side monocytes of calcified regions (Figure 7I). This suggests that the high expression of the two genes may be associated with the immune cell activation identified in the bulk-RNA data.

### Impact of fibro-calcification score on cell functions in aortic calcification

**Figure 8:**
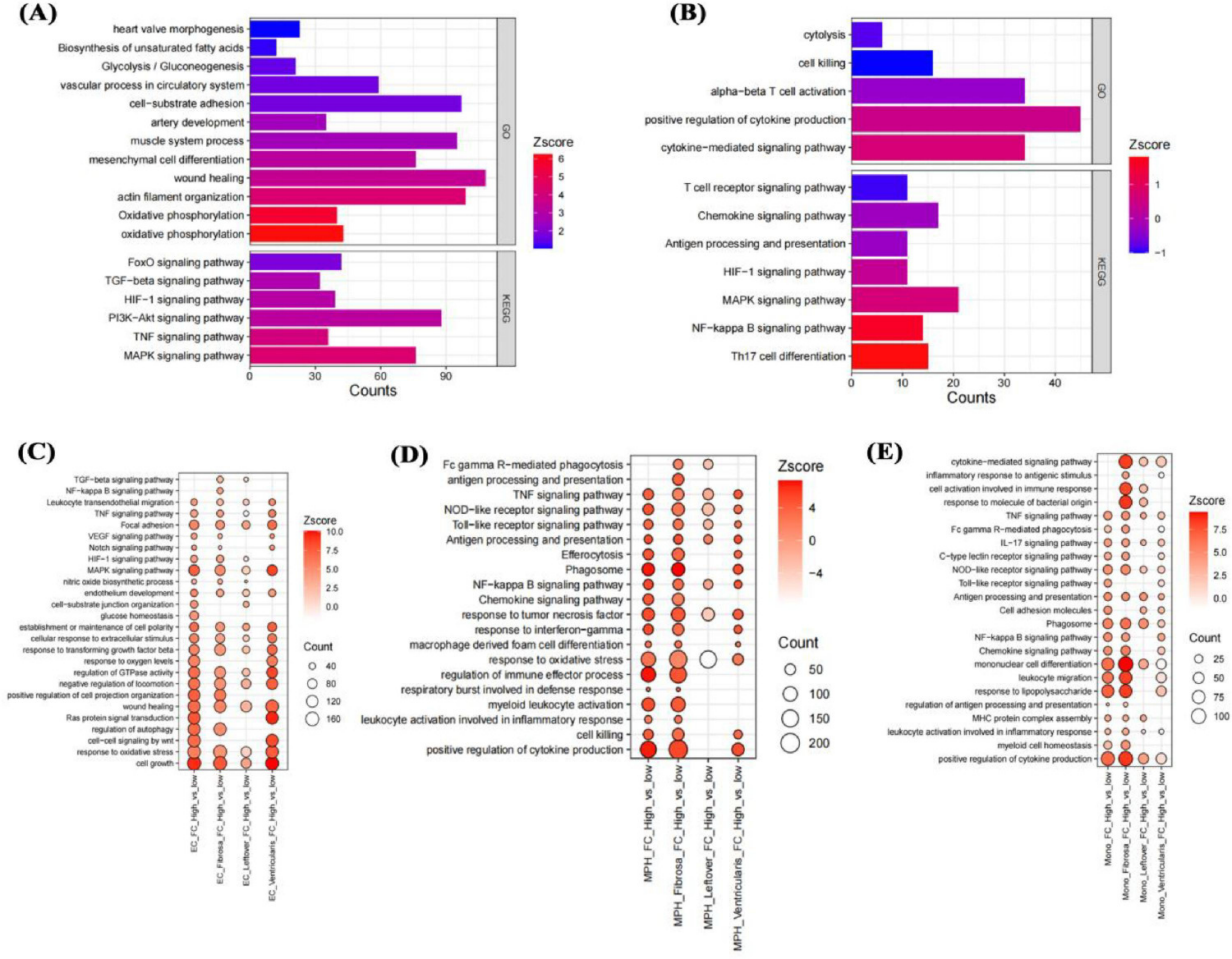
Impact of fibro-calcification score on cell functions. Effects of FC score on smooth muscle cell and T cell functions in the fibrosa layer **(A,B)**; effects on endothelial cell, macrophage, and monocyte functions in the fibrosa layer, mixed remaining layer, and ventricular layer **(C–E)**.

To clarify the impact of fibrosis on different cells, the study conducted a systematic analysis of 5 cell types in different regions separately. In smooth muscle cells, higher FC scores were associated with significant changes in cardiac valve morphology, glycolysis, damage repair, TGF-β signaling pathway, and HIF-1 signaling pathway compared with lower FC scores (Figure 8A). In T cells with higher FC scores, cytokine production, T cell receptor signaling pathway, NF-κB signaling pathway, and HIF-1 signaling pathway were significantly altered compared with lower scores (Figure 8B). In different regions of the aortic valve, highly calcified endothelial cells showed significant upregulation of TNF signaling pathway, MAPK signaling pathway, endothelial cell development, and response to oxidative stress (Figure 8C). In macrophages, the highly calcified fibrosa layer showed stronger proinflammatory signals (Figure 8D). Similar to macrophages, monocytes in the highly calcified fibrosa layer also showed enhanced proinflammatory signals (Figure 8E), possibly an important factor in their differentiation into macrophages.

### Impact of genes on functions of different cells in aortic calcification

**Figure 9:**
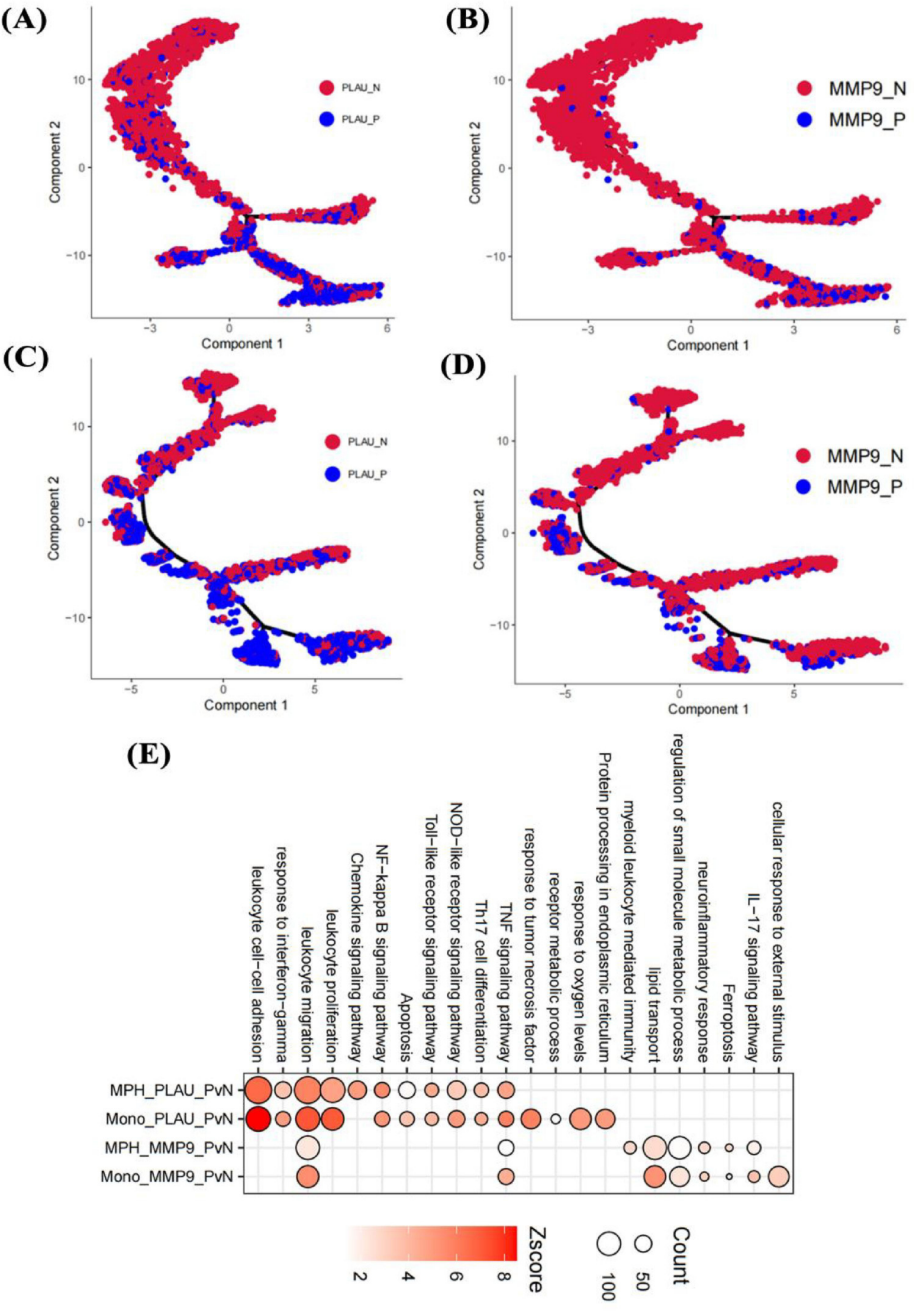
Effects of genes on cell pseudotime and functions. Impact of PLAU and MMP9 on differentiation trajectories of monocytes **(A,B)** and macrophages **(C,D)**; effects on functions of endothelial cells, monocytes, and macrophages in the fibrosa layer **(E)**.

Given the high expression of PLAU and MMP9 in macrophages and monocytes of highly calcified regions, pseudotime analysis was used to clarify their roles. In monocytes, initial positions of the differentiation trajectory were mainly cells not expressing PLAU and MMP9, while terminal positions were cells expressing both genes (Figures 9A,B). Similar to monocytes, initial-position cells in macrophage differentiation trajectories did not express PLAU and MMP9, while terminal cells mainly expressed these genes (Figures 9C,D). Compared with cells not expressing PLAU, PLAU-expressing cells had enhanced chemotaxis, migration, and inflammatory responses (Figure 9E). Compared with cells not expressing MMP9, MMP9-expressing cells showed enhanced lipid transport and small-molecule metabolism (Figure 9E). Therefore, these two genes may play different roles in the functions of monocytes and macrophages.

## Discussion

AS is considered an active disease, mainly divided into initiation and propagation stages (30). The former is primarily characterized by endothelial cell injury and low-density lipoprotein accumulation in the valve, stimulating monocyte infiltration and differentiation into macrophages (31). Early aortic valve macrophages recruit other immune cells to further exacerbate endothelial injury, consistent with the enhanced inflammatory response in early aortic sclerosis found in this study. Meanwhile, enhanced TNF signaling pathway and oxidative stress response in endothelial cells of highly calcified aortic valves also reflect immune cell stimulation. In the propagation stage, valve fibrosis and calcification are key triggers for AS (32). In different structural heart diseases, fibrosis is an important driver of heart failure (33). This study found significant increases in fibrosis-related genes and ECM formation in AS patients through two RNA-seq datasets and proteomic sequencing. Current research indicates that hypoxia-induced glycolysis is an important factor in disease deterioration, widely involved in tissue fibrosis (34). This study also found activation of the HIF-1 signaling pathway in the transcriptome, proteome, and single-cell transcriptome of AS patients, suggesting it may be a potential therapeutic target for AS.

Most current studies primarily use transcriptomics to identify AS pathogenic genes, but since proteins are the direct executors of biological functions, this study combined transcriptomics from public databases with proteomic sequencing. At the transcriptional level of aortic valve tissue, DEGs and biological functions of aortic calcification were analyzed independently, and genes from important modules were intersected to obtain robust candidates. Numerous studies have shown that MMPs are involved in ECM formation (35), which was also observed in the AS transcriptome. Consistent with current views, this study found that immune cells characterized by upregulated chemokines and proinflammatory factors actively participate in AS progression, indicating an important role of immune cells in AS fibrosis. At the protein level of aortic valve tissue, calcified AS patients showed enhanced fibrosis and inflammation. Identification of 16 hub genes through multi-algorithms revealed that 13/16 had high diagnostic efficacy, promising for histopathological diagnosis. Surprisingly, only 4/16 genes showed consistent mRNA and protein levels, but this partially avoids the limitations of single-omics analysis. The study finally found that MMP9 and PLAU showed significantly increased mRNA and protein levels after AS occurrence. Notably, due to the difficulty in collecting samples from patients with AS, this study used 8 samples per group for proteomic sequencing, which to a certain extent increases the risk of false positives. To address this limitation, verification was performed on patient tissues with 3 samples per group. It is worth noting that the heterogeneity in smoking, alcohol consumption, and coronary artery disease among the patients included in the study for proteomic sequencing may have a potential impact on the expression profiles. For instance, patients with AS group had lower rates of smoking and alcohol consumption, which might have resulted in the absence of observations related to inflammatory factors and oxidative stress in their protein expression profiles (36). Additionally, patients with AS group had a higher prevalence of a history of coronary artery disease, which could have led to an overemphasis on biological responses associated with ischemia and hypoxia (37). However, since the identified hub genes were not directly associated with reduced inflammation-oxidative stress or enhanced ischemia-hypoxia, the adverse effects caused by these baseline differences were significantly mitigated.

Tissue-level transcriptomics and proteomics are widely used for biomarker development, but their mechanistic research remains to be further explored. Current studies have found that MMP9 and PLAU are involved in extracellular matrix formation, mainly produced by fibroblasts (35, 38). This study, through single-cell transcriptomics, found that MMP9 and PLAU are not highly expressed in smooth muscle cells and T cells of highly calcified regions but are mainly associated with endothelial cells, monocytes, and macrophages. This suggests that these two genes may be involved in endothelial cell injury and early immune activation of monocyte-macrophages in aortic calcification. Using pseudotime analysis, we verified that the expression of MMP9 and PLAU is involved in the differentiation process of monocytes and macrophages. Meanwhile, lipid metabolism is a hallmark of activated macrophages—a characteristic also observed in the MMP9^+^ monocyte/macrophage population identified in the present study. Previous studies (39) have established that upon migrating to the valvular region, monocytes differentiate into macrophages; these macrophages then secrete proinflammatory factors to recruit additional monocytes. This pathological cascade may be linked to the enhanced chemotaxis of PLAU^+^ monocytes and the amplified inflammatory response of MMP9^+^ macrophages, as documented in our research. Consequently, MMP9 and PLAU hold promise as potential synergistic targets for anti-inflammatory interventions against AS. Notably, MMP9 and PLAU display higher expression in monocytes residing within the calcified ventricularis layer—a valvular region exposed to substantial mechanical stress. This observation suggests that the expression of these two genes may be modulated by mechanical stress, thereby indirectly contributing to valvular calcification under high-pressure conditions. It further implies the existence of a more robust monocyte-macrophage activation loop within the ventricularis layer, which could exacerbate the progression of AS-related valvular pathology.

An observational study showed that in pediatric patients, circulating MMPs, including MMP9, can be used to predict aortic dilation associated with bicuspid aortic valves (40). Given that both aortic valve calcification and dilation present an inflammatory phenotype, MMP9 may also serve as a marker for the progression of valvular calcification. PLAU is a chronic inflammatory marker; studies using GWAS analysis have revealed that it exhibits high variability in blood samples, suggesting it could be a crucial observation indicator (41). However, further research is needed to explore its role in patients with calcific aortic stenosis. Since there are certain differences in smoking, alcohol consumption, and other aspects between the two groups of patients enrolled in this study, a comprehensive assessment combining imaging examinations and other biomarkers is required in clinical application to improve the accuracy of diagnosis.

In conclusion, this study screened 16 hub genes from the proteome that can assist in AS diagnosis. Combining bulk RNA-seq and scRNA-seq, it was found that MMP9 and PLAU are mainly related to the immune activation of monocytes and macrophages in aortic valve calcification, providing new insights for early AS treatment.

## Data Availability

The original contributions presented in the study are publicly available. This data can be found here: .Bulk-RNAseq (accession numbers: GSE51472 and GSE12644) and scRNAseq (accession numbers: GSE220774) data are available in GEO database. The proteomics data has been uploaded to the iProX database, with the access ID IPX0012811001.

## References

[1] Nkomo VT Gardin JM Skelton TN Gottdiener JS Scott CG Enriquez-Sarano M. Burden of valvular heart diseases: a population-based study. Lancet. (2006) 368(9540):1005–11. 10.1016/S0140-6736(06)69208-8 16980116

[2] Otto CM Nishimura RA Bonow RO Carabello BA Erwin JP Gentile F 2020 ACC/AHA guideline for the management of patients with valvular heart disease: a report of the American College of Cardiology/American Heart Association joint committee on clinical practice guidelines. J Am Coll Cardiol. (2021) 77(4):e25–197. 10.1016/j.jacc.2020.11.018 33342586

[3] Blaser MC Bäck M Lüscher TF Aikawa E. Calcific aortic stenosis: omics-based target discovery and therapy development. Eur Heart J. (2025) 46(7):620–34. 10.1093/eurheartj/ehae829 39656785 PMC11825147

[4] Mathieu P Bossé Y Huggins GS Corte AD Pibarot P Michelena HI The pathology and pathobiology of bicuspid aortic valve: state of the art and novel research perspectives. J Pathol Clin Res. (2015) 1(4):195–206. 10.1002/cjp2.21 27499904 PMC4939890

[5] Rajamannan NM Evans FJ Aikawa E Grande-Allen KJ Demer LL Heistad DD Calcific aortic valve disease: not simply a degenerative process: a review and agenda for research from the national heart and lung and blood institute aortic stenosis working group. Executive summary: calcific aortic valve disease-2011 update. Circulation. (2011) 124(16):1783–91. 10.1161/CIRCULATIONAHA.110.006767 22007101 PMC3306614

[6] Greenberg HZE Zhao G Shah AM Zhang M. Role of oxidative stress in calcific aortic valve disease and its therapeutic implications. Cardiovasc Res. (2022) 118(6):1433–51. 10.1093/cvr/cvab142 33881501 PMC9074995

[7] Kadoglou NP Stasinopoulou M Gkougkoudi E Christodoulou E Kostomitsopoulos N Valsami G. The complementary effects of dabigatran etexilate and exercise training on the development and stability of the atherosclerotic lesions in diabetic ApoE knockout mice. Pharmaceuticals (Basel). (2023) 16(10):1396. 10.3390/ph16101396 37895867 PMC10609840

[8] Gomes AV. Spatiotemporal multi-omics-derived atlas of calcific aortic valve disease. Circulation. (2018) 138(4):394–6. 10.1161/CIRCULATIONAHA.118.035431 30571374 PMC6309777

[9] Gravina M Casavecchia G Manuppelli V Totaro A Macarini L Di Biase M Mitral annular calcification: can CMR be useful in identifying caseous necrosis? Interv Med Appl Sci. (2019) 11(1):71–3. 10.1556/1646.10.2018.47 32148907 PMC7044563

[10] Zhong G Su S Li J Zhao H Hu D Chen J Activation of piezo1 promotes osteogenic differentiation of aortic valve interstitial cell through YAP-dependent glutaminolysis. Sci Adv. (2023) 9(22):eadg0478. 10.1126/sciadv.adg0478 37267365 PMC10413650

[11] Zeng P Yang J Liu L Yang X Yao Z Ma C ERK1/2 inhibition reduces vascular calcification by activating miR-126-3p-DKK1/LRP6 pathway. Theranostics. (2021) 11(3):1129–46. 10.7150/thno.49771 33391525 PMC7738895

[12] Ohukainen P Syväranta S Näpänkangas J Rajamäki K Taskinen P Peltonen T MicroRNA-125b and chemokine CCL4 expression are associated with calcific aortic valve disease. Ann Med. (2015) 47(5):423–9. 10.3109/07853890.2015.1059955 26203686

[13] Bossé Y Miqdad A Fournier D Pépin A Pibarot P Mathieu P. Refining molecular pathways leading to calcific aortic valve stenosis by studying gene expression profile of normal and calcified stenotic human aortic valves. Circ Cardiovasc Genet. (2009) 2(5):489–98. 10.1161/CIRCGENETICS.108.820795 20031625

[14] Robinson MD McCarthy DJ Smyth GK. Edger: a bioconductor package for differential expression analysis of digital gene expression data. Bioinformatics. (2010) 26(1):139–40. 10.1093/bioinformatics/btp616 19910308 PMC2796818

[15] Liberzon A Birger C Thorvaldsdóttir H Ghandi M Mesirov J Tamayo P. The molecular signatures database (MSigDB) hallmark gene set collection. Cell Syst. (2015) 1(6):417–25. 10.1016/j.cels.2015.12.004 26771021 PMC4707969

[16] Szklarczyk D Gable AL Lyon D Junge A Wyder S Huerta-Cepas J STRING V11: protein-protein association networks with increased coverage, supporting functional discovery in genome-wide experimental datasets. Nucleic Acids Res. (2019) 47(D1):D607–13. 10.1093/nar/gky1131 30476243 PMC6323986

[17] Shannon P Markiel A Ozier O Baliga NS Wang JT Ramage D Cytoscape: a software environment for integrated models of biomolecular interaction networks. Genome Res. (2003) 13(11):2498–504. 10.1101/gr.1239303 14597658 PMC403769

[18] Yu G Wang L-G Han Y He Q-Y. Clusterprofiler: an R package for comparing biological themes among gene clusters. OMICS. (2012) 16(5):284–7. 10.1089/omi.2011.0118 22455463 PMC3339379

[19] Tang D Chen M Huang X Zhang G Zeng L Zhang G SRplot: a free online platform for data visualization and graphing. PLoS One. (2023) 18(11):e0294236. 10.1371/journal.pone.0294236 37943830 PMC10635526

[20] Bader GD Hogue CW. An automated method for finding molecular complexes in large protein interaction networks. BMC Bioinform. (2003) 4:2. 10.1186/1471-2105-4-2 PMC149346 12525261

[21] Chin C-H Chen S-H Wu H-H Ho C-W Ko M-T Lin C-Y. Cytohubba: identifying hub objects and sub-networks from complex interactome. BMC Syst Biol. (2014) 8(Suppl 4):S11. 10.1186/1752-0509-8-S4-S11 25521941 PMC4290687

[22] Lex A Gehlenborg N Strobelt H Vuillemot R Pfister H. Upset: visualization of intersecting sets. IEEE Trans Vis Comput Graph. (2014) 20(12):1983–92. 10.1109/TVCG.2014.2346248 26356912 PMC4720993

[23] Han H Shim H Shin D Shim JE Ko Y Shin J TRRUST: a reference database of human transcriptional regulatory interactions. Sci Rep. (2015) 5:11432. 10.1038/srep11432 26066708 PMC4464350

[24] Deng S Zhang H Zhu K Li X Ye Y Li R M6A2Target: a comprehensive database for targets of m6A writers, erasers and readers. Brief Bioinform. (2021) 22(3):bbaa055. 10.1093/bib/bbaa055 32392583

[25] Villa-Roel N Park C Andueza A Baek KI Su A Blaser MC Side- and disease-dependent changes in human aortic valve cell population and transcriptomic heterogeneity determined by single-cell RNA sequencing. Genes (Basel). (2024) 15(12):1623. 10.3390/genes15121623 39766890 PMC11675841

[26] Butler A Hoffman P Smibert P Papalexi E Satija R. Integrating single-cell transcriptomic data across different conditions, technologies, and species. Nat Biotechnol. (2018) 36(5):411–20. 10.1038/nbt.4096 29608179 PMC6700744

[27] Aran D Looney AP Liu L Wu E Fong V Hsu A Reference-based analysis of lung single-cell sequencing reveals a transitional profibrotic macrophage. Nat Immunol. (2019) 20(2):163–72. 10.1038/s41590-018-0276-y 30643263 PMC6340744

[28] Hu C Li T Xu Y Zhang X Li F Bai J Cellmarker 2.0: an updated database of manually curated cell markers in human/mouse and web tools based on scRNA-Seq data. Nucleic Acids Res. (2023) 51(D1):D870–6. 10.1093/nar/gkac947 36300619 PMC9825416

[29] Cao J Packer JS Ramani V Cusanovich DA Huynh C Daza R Comprehensive single-cell transcriptional profiling of a multicellular organism. Science. (2017) 357(6352):661–7. 10.1126/science.aam8940 28818938 PMC5894354

[30] Lindman BR Clavel M-A Mathieu P Iung B Lancellotti P Otto CM Calcific aortic stenosis. Nat Rev Dis Primers. (2016) 2:16006. 10.1038/nrdp.2016.6 27188578 PMC5127286

[31] Peeters FECM Meex SJR Dweck MR Aikawa E Crijns HJGM Schurgers LJ Calcific aortic valve stenosis: hard disease in the heart: a biomolecular approach towards diagnosis and treatment. Eur Heart J. (2018) 39(28):2618–24. 10.1093/eurheartj/ehx653 29136138 PMC6055545

[32] Grim JC Aguado BA Vogt BJ Batan D Andrichik CL Schroeder ME Secreted factors from proinflammatory macrophages promote an osteoblast-like phenotype in valvular interstitial cells. Arterioscler Thromb Vasc Biol. (2020) 40(11):e296–308. 10.1161/ATVBAHA.120.315261 32938214 PMC7578003

[33] Frangogiannis NG. Cardiac fibrosis. Cardiovasc Res. (2021) 117(6):1450–88. 10.1093/cvr/cvaa324 33135058 PMC8152700

[34] Aventaggiato M Barreca F Sansone L Pellegrini L Russo MA Cordani M Sirtuins and hypoxia in EMT control. Pharmaceuticals (Basel). (2022) 15(6):737. 10.3390/ph15060737 35745656 PMC9228842

[35] Lee C-J Jang T-Y Jeon S-E Yun H-J Cho Y-H Lim D-Y The dysadherin/MMP9 axis modifies the extracellular matrix to accelerate colorectal cancer progression. Nat Commun. (2024) 15(1):10422. 10.1038/s41467-024-54920-9 39613801 PMC11607440

[36] Caliri AW Tommasi S Besaratinia A. Relationships among smoking, oxidative stress, inflammation, macromolecular damage, and cancer. Mutat Res Rev Mutat Res. (2021) 787:108365. 10.1016/j.mrrev.2021.108365 34083039 PMC8287787

[37] Pan J Zhang L Li D Li Y Lu M Hu Y Hypoxia-inducible factor-1: regulatory mechanisms and drug therapy in myocardial infarction. Eur J Pharmacol. (2024) 963:176277. 10.1016/j.ejphar.2023.176277 38123007

[38] Fang L Che Y Zhang C Huang J Lei Y Lu Z PLAU Directs conversion of fibroblasts to inflammatory cancer-associated fibroblasts, promoting esophageal squamous cell carcinoma progression via uPAR/akt/NF-κB/IL8 pathway. Cell Death Discov. (2021) 7(1):32. 10.1038/s41420-021-00410-6 33574243 PMC7878926

[39] Zhang P The E Luo Z Zhai Y Yao Q Ao L Pro-inflammatory mediators released by activated monocytes promote aortic valve fibrocalcific activity. Mol Med. (2022) 28(1):5. 10.1186/s10020-022-00433-4 35062861 PMC8780233

[40] Făgărășan A Săsăran MO Gozar L Toma D Șuteu C Ghiragosian-Rusu S Circulating matrix metalloproteinases for prediction of aortic dilatation in children with bicuspid aortic valve: a single-center, observational study. Int J Mol Sci. (2024) 25(19):10538. 10.3390/ijms251910538 39408865 PMC11476682

[41] Dowsett J Ferkingstad E Rasmussen LJH Thørner LW Magnússon MK Sugden K Eleven genomic loci affect plasma levels of chronic inflammation marker soluble urokinase-type plasminogen activator receptor. Commun Biol. (2021) 4(1):655. 10.1038/s42003-021-02144-8 34079037 PMC8172928

