# Supplementary material for: Integrated multi-omics analysis reveals key hub genes and mechanisms in calcific aortic stenosis
Source: Front Cardiovasc Med. 2025 Nov 14;12:1640014. doi: 10.3389/fcvm.2025.1640014 (PMC12660111; doi:10.3389/fcvm.2025.1640014)
Supplement: Supplementary file 1 [file Table1.pdf]

Supplementary table 1 Comparison of basic information of patients

Variable	Control	AS	t/z
Age	63±3	66±3	-1.994
Gender			0.000
Male	5（62.5%）	5（62.5%）	
Female	3（37.5%）	3（37.5%）	
Smoking	5（62.5%）	1（12.5%）	2.400
Alcohol consumption	5（62.5%）	1（12.5%）	2.400
Hypertension	4（50.0%）	3（37.5%）	0.000
Diabetes mellitus	8（100%）	8（100%）	—
Coronary heart disease	0（0%）	3（37.5%）	1.641
Atrial fibrillation	0（0%）	1（12.5%）	0.000
Pacemaker	8（100%）	8（100%）	—
Statins	2（25%）	3（37.5%）	0.000
Aspirin	1（12.5%）	3（37.5%）	0.333
ACEI/ARB	6(75.0%)	4（50.0%）	0.267
β-blocker	0（0%）	2（25%）	0.571
BMI	21.88±4.00	20.32±2.58	0.924
Systolic blood pressure (mmHg)	134±18	109±12	3.27
Diastolic blood pressure (mmHg)	66±15	65±10	0.06
Annulus (mm)	25.13±3.48	22.88±3.98	1.203
Left atrium (mm)	42.38±3.82	40.5±6.12	0.736
End-diastolic diameter (mm)	66.13±11.66	55.13±10.92	1.948
End-systolic diameter (mm)	45.38±9.27	38.13±9.64	1.533
Interventricular septum (mm)	10.5±2.507	11.88±2.100	-1.189
Left ventricular posterior wall (mm)	10.38±2.264	11.25±1.909	-0.836
AR m/s (Aortic forward blood flow velocity)	2.23±0.40	4.31±1.03	-5.284
SV(ml)	133.42±49.53	92.98±39.22	1.811
FS(%)	31.60±4.50	30.52±5.19	0.444
EF(%)	58.17±6.86	57.68±8.48	0.127
Alanine aminotransferase (U/L)	25.85±15.09	24.52±7.67	0.222
Aspartate aminotransferase (U/L)	24.64±8.70	29.23±9.08	-1.033
Cholinesterase (U/L)	7705.99±866.27	7358.93±861.33	0.804
Uric acid (μmol/L)	304.99±97.83	328.78±106.74	-0.465
Creatinine (μmol/L)	72.6±13.59	74.26±22.90	-0.177
BNP (pg/mL)	926.75±518.69	1000.8±597.76	-0.265
High-density lipoprotein (mmol/L)	0.97±0.20	1.11±0.23	-1.304

Low-density lipoprotein cholesterol (mmol/L)	2.05±0.62	2.82±0.83	-2.116
Triglyceride (mmol/L)	1.40±0.43	1.40±0.42	-0.012
Total cholesterol (mmol/L)	3.43±0.51	4.51±0.95	-2.832
D-dimer (mg/L)	0.40±0.18	0.32±0.19	0.787
Glucose (mmol/L)	4.74±0.63	4.89±0.44	-0.579
Albumin (g/L)	40.77±3.86	39.58±2.80	0.707
Potassium (mmol/L)	3.71±0.41	4.01±0.51	-1.298
Sodium (mmol/L)	142.22±2.64	140.51±2.31	1.381
leukocyte count (10^9/L)	6.32±1.74	7.38±3.02	-0.860
Erythrocyte (10^9/L)	4.64±0.51	4.28±0.49	1.419
Hemoglobin content(g/L)	141±9.94	126.25±18.46	1.99
Platelets(10^9/L)	208.38±55.28	262.38±98.83	-1.349
Neutrophil count(10^9/L)	3.52±1.18	4.84±2.48	-1.363
Lymphocyte count(10^9/L)	2.16±0.75	1.72±0.51	1.384
Basophil count(10^9/L)	0.05±0.02	0.04±0.02	0.540
C-reactive protein(mg/L)	12.00±5.76	11.71±5.50	0.107
Homocysteine(ummol/L)	15.25±2.29	17.37±3.67	-1.386
Aorta (sinus) (mm)	46.5(34.75,54.25)	34(30.5,36)	-1.947
Right ventricular outflow tract (mm)	30(30,31)	29(27,30)	-2.247
Right ventricle (mm)	20(19.25,20)	20(19.25,20)	-0.387
Right atrium (mm)	35(34,36.75)	34.5(33,35.75)	-0.749
Pulmonary artery (mm)	25(22.25,25)	23(20,25)	-0.936
CO(l/min)	9.75(8.97,11.58)	43.79(6.08,97)	-0.420
Effective orifice area	4.80(4.63,4.88)	0.78(0.55,0.80)	-3.381
Alkaline phosphatase (U/L)	70.72(66.61,73.74)	69(57.24,93.61)	-0.263
γ-glutamyl transpeptidase (U/L)	21.26(17.25,37.19)	39.01(26.30,60.17)	-1.785
Blood urea nitrogen (mmol/L)	6.21(6.10,8.56)	7.62(6.37,9.14)	-1.05
Monocyte count (10^9/L)	0.55(0.37,0.68)	0.48(0.46,0.71)	-0.105
Eosinophil count (10^9/L)	0.13(0.10,0.23)	0.16(0.07,0.39)	-0.21
